# Natural life cycle of *Versteria cuja* (Taeniidae) in Argentina and histopathology of metacestodiasis in intermediate hosts

**DOI:** 10.1017/S0031182023000215

**Published:** 2023-05

**Authors:** Estefanía Bagnato, Francisco Acuña, Federico Brook, Gabriel Mario Martin, Claudio Gustavo Barbeito, María Celina Digiani

**Affiliations:** 1 Laboratorio de Investigaciones en Evolución y Biodiversidad (LIEB), Facultad de Ciencias Naturales Sede Esquel, Universidad Nacional de la Patagonia ‘San Juan Bosco’ (UNPSJB) Ruta Nacional N° 259, 16.4 Km, (9200) Esquel, Chubut, Argentina; 2 Laboratorio de Histología y Embriología Descriptiva, Experimental y Comparada, Facultad de Ciencias Veterinarias, Universidad Nacional de La Plata, 60 y 118, s/n, (1900), La Plata, Buenos Aires, Argentina; 3 CONICET-CCT La Plata, Calle 8 Nº 1467, (1904) La Plata, Buenos Aires, Argentina; 4 Centro de Investigación Esquel de Montaña y Estepa Patagónica (CIEMEP), Consejo Nacional de Investigaciones Científicas y Técnicas (CONICET)-UNPSJB, General Roca 780, (9200) Esquel, Chubut, Argentina; 5 División Zoología Invertebrados, Facultad de Ciencias Naturales y Museo, Universidad Nacional de La Plata, Paseo del Bosque s/n, (1900) La Plata, Buenos Aires, Argentina

**Keywords:** Argentina, cox1, *Ctenomys*, histopathology, life cycle, Mustelidae, rodents, *Versteria*

## Abstract

Using morphological and molecular studies, the life cycle of *Versteria cuja* (Cestoda: Taeniidae) was elucidated, involving subterranean rodents (Ctenomyidae) as intermediate hosts, and the lesser grison, *Galictis cuja* (Mustelidae), as definitive host. Metacestodes (cysticerci and polycephalic larvae) were found mainly in the liver but also in spleen, pancreas, lungs and small intestine of 2 species of tuco-tucos (*Ctenomys* spp.) from Chubut, Argentina. Identity of the metacestodes with the adult was based primarily on the number, size and shape of rostellar hooks: 40–48 hooks in 2 rows, particularly small (10–16 *μ*m total length by 6–10 *μ*m wide), composed of handle, blade and guard with characteristic shapes. Genetic analysis (cox1 gen mtDNA) performed on metacestodes from both intermediate hosts corroborated their conspecificity with adults of *V. cuja* from lesser grisons in the same locality. Histopathological study showed the hepatic parenchyma altered by the presence of cysts containing larvae, each surrounded by a capsule of connective tissue with inflammatory infiltrate, atrophied hepatocytes and an increase of bile ducts. In the lung, in addition to the cysts, dilated alveoli, oedema and hyperaemic blood vessels were observed. This is the first report of a natural life cycle of a *Versteria* species from South America. It shows strong similarities with that described for a North American zoonotic lineage of *Versteria*, confirming a close relationship between *V. cuja* and this North American lineage, as previously demonstrated by molecular studies. Consequently, the zoonotic potential of *V. cuja* should not be disregarded.

## Graphical abstract

**Figure:**
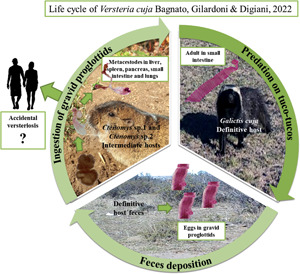

## Introduction

Taeniid species have a worldwide distribution and are of medical and veterinary importance because adult tapeworms are responsible for intestinal infections, whereas metacestodes are responsible for systemic infections in domestic and wild animals, and in humans (Deplazes *et al*., 2019). In taeniid life cycles, members of the order Carnivora mainly act as definitive hosts whereas their prey, largely belonging to the orders Rodentia, Lagomorpha and Artiodactyla, act as intermediate hosts (Loos-Frank, 2000; Hoberg, 2006). Within the family, the genus *Versteria* Nakao, Lavikainen, Iwaki, Konyaev, Oku, Okamoto and Ito, 2013 includes 3 species: *Versteria mustelae* (Gmelin, 1790) (type-species), with an Holarctic distribution; *Versteria brachyacantha* (Baer and Fain, 1951) described from Africa (current Rwanda); and *Versteria cuja* Bagnato, Gilardoni and Digiani, 2022 from Argentina. Recent studies suggested that: (1) *V. mustelae* could represent a species complex, in which at least 4 different lineages can be identified (Western Europe, Siberia /Japan, China and USA), and (2) there are 2 different lineages of *Versteria* in the Nearctic: one related to the Palearctic *V. mustelae* and another one, not yet formally described but characterized molecularly, known as ‘zoonotic’ *Versteria* sp. and responsible for fatal and/or severe infections by metacestodes in wildlife, captive primates and immunosuppressed people (Goldberg *et al*., 2014; Lee *et al*., 2016; Deplazes *et al*., 2019; Niedringhaus *et al*., 2021). Some of the older reports of *Taenia mustelae* from North America (e.g. Skinker, 1935; Locker, 1955; Freeman, 1956) could be either species.

*Versteria cuja* seems to be related to the Nearctic *Versteria* sp. (Bagnato *et al*., 2022) and the conspecificity of both taxa cannot be ruled out. Consequently, *V. cuja* is of importance in view of its zoonotic potential in this region. *Versteria cuja* uses the lesser grison, *Galictis cuja* (Molina) as definitive host, a native mustelid which occupies a wide variety of habitats (Larivière and Jennings, 2009), and whose diet is mostly composed of small to medium sized vertebrates (e.g. rodents, hares) (Chébez *et al*., 2014). Particularly in Chubut province (Argentina), near Esquel and Trevelin cities (the type locality of *V. cuja*), the lesser grisons mainly feed on rodents, including tuco-tucos *Ctenomys* Blainville (Rodentia: Ctenomyidae) (Delibes *et al*., 2003; G. M. Martin, pers. obs.). In the neighbourhood of the type locality of *V. cuja*, and in the frame of a study conducted by mammalogists (Doctoral Fellowship project of F. Brook), 37 specimens of tuco-tucos of different species were captured and examined for parasites. Two of the rodents examined (*Ctenomys* sp. 1 and *Ctenomys* sp. 2) harboured metacestodes in different organs. Molecular analyses conducted on these metacestodes corroborated their conspecificity with the adults of *V. cuja* found in lesser grisons in the type locality. The aim of this paper is to shed light on the 2-host life cycle of *V. cuja*, providing a morphological and molecular characterization of the metacestodes found, as well as a histopathological description of the metacestodiasis affecting the liver and the lungs of the intermediate hosts.

## Materials and methods

### Study area and sample collection

Four individuals of *Ctenomys* sp. 1 were collected near Terraplén Lagoon (42.96°S, 71.49°W) in April 2019, Chubut province, Argentina. The habitat is a transitional environment between the Subantarctic beech-tree forest to the west and the Patagonian steppe to the east. One individual of *Ctenomys* sp. 2 was collected near Talagapa (42.16°S, 68.17°W) (Patagonian steppe) in November 2021. The names ‘*Ctenomys* sp. 1’ and ‘*Ctenomys* sp. 2’ represent 2 different, undescribed species, studied as part of the Doctoral work of F. Brook and still unpublished. One specimen of *Ctenomys haigi* Thomas collected near Esquel Airport ‘Brigadier General Antonio Parodi’ (42.87°S, 71.13°W) in March 2022 was available for histological comparison of normal liver tissue. All captures were made under permits provided by the *Dirección Fauna y Flora Silvestre* from the *Ministerio de Agricultura, Ganadería, Industria y Comercio del Chubut* (Resolutions N° 1468/2019, N° 404/2021). Specimens were caught using Oneida Victor N° 0 traps with rubber covers and were euthanized by cervical dislocation (Sikes *et al*., 2011; Brook *et al*., 2022). Tuco-tucos from Terraplén Lagoon were necropsied fresh and the *Ctenomys* sp. 2 from Talagapa was fixed in 96% ethanol before examination. All of them were inspected for endoparasites under a Leica EZ4 stereomicroscope (Leica, Wetzlar, Germany). The gastrointestinal tract was separated into oesophagus, stomach, caecum and intestine. The body cavity, liver, pancreas, spleen, gall bladder, gonads, lungs, heart and kidneys were also examined for parasites.

### Parasitological study

Most metacestodes from *Ctenomys* sp. 1 were fixed in 4% formalin/distilled water (after washing with physiological solution), and preserved and stored in 70% ethanol; some others were stored directly in absolute ethanol for molecular analysis. Metacestodes from *Ctenomys* sp. 2 were fixed and preserved in 96% ethanol. Specimens designated for morphological study were stained with Semichon's acetic carmine orLangeron's carmine, dehydrated through an ethanol series, cleared in eugenol and mounted in Canada balsam for examination under a light microscope Leica DM500 (Leica, Wetzlar, Germany). To observe the rostellum and rostellar hooks in detail, rostella from several metacestodes were dissected and mounted between slides and coverslips in Hoyer's fluid and allowed to dry. Measurements and photographs were taken with a Leica ICC50W camera with software connected to the microscope. Measurements are given in micrometres (*μ*m) as range, followed by mean in parentheses, unless otherwise stated. Prevalence and mean intensity of metacestodes infection in *Ctenomys* sp. 1 were calculated following Bush *et al*. (1997). Mounted vouchers of metacestodes of *V. cuja* were deposited in the Helminthological Collection of the Museo de La Plata (MLP-He), La Plata, Argentina. Specimens of *Ctenomys* sp. 1, *Ctenomys* sp. 2 and *C. haigi* were deposited in the Mammal Collection of the *Laboratorio de Investigaciones en Evolución y Biodiversidad* (LIEB-M), Esquel, Argentina.

### Statistical analysis

A 1-way analysis of variance was performed (factor: intermediate host species) using the R software and plyr package (R Core Team, 2023) in R Studio (RStudio Team, 2023) for summary measurements of the metacestodes (mean ± standard error), shown in Table 1.

**Table 1.** Comparison of some measured characters (in micrometres) of *Versteria cuja* metacestodes (mono- and polycephalic forms) between *Ctenomys* sp. 1 from Terraplén Lagoon and *Ctenomys* sp. 2 from Talagapa, Chubut province, Argentina, using one-way analysis of variance (ANOVA)

Characters	Mean ± s.e.	Mean ± s.e.	*P* value
Monocephalic forms	*Ctenomys* sp. 1	*Ctenomys* sp. 2	
Total length	3280 ± 221	3260 ± 325	0.97
Maximum width	1278 ± 58	1419 ± 113	0.30
Scolex width	279 ± 16	262 ± 8	0.56
Rostellum diameter	58 ± 12	55 ± 3	0.88
Suckers diameter	105 ± 6	118 ± 10	0.24
Neck length	433 ± 40	611 ± 25	0.009**
Neck width	286 ± 31	355 ± 32	0.19
Polycephalic forms			
Number of scoleces per bladder	3 ± 0.3	3 ± 0.3	0.85
Total length	8322 ± 954	4682 ± 423	0.0004***
Maximum width	2518 ± 427	3205 ± 291	0.18
Exogenous buds length	4762 ± 836	2094 ± 228	0.0007***
Exogenous buds width	1140 ± 101	1325 ± 98	0.23
Central bladder length	1943 ± 1041	1956 ± 212	0.98
Central bladder width	843 ± 203	2204 ± 343	0.06
Scolex width	286 ± 18	278 ± 18	0.78
Rostellum diameter	63 ± 8	41 ± 7	0.08
Suckers diameter	112 ± 3	131 ± 7	0.07
Neck length	520 ± 47	760 ± 33	0.0002***
Neck width	271 ± 29	370 ± 26	0.02*

s.e., standard error.

Asterisks for *P* levels (*P < 0.05, **P < 0.01, ***P < 0.001).

### DNA extraction, amplification and sequencing

Eight sequences were generated from a mitochondrial DNA region, cytochrome c oxidase subunit 1 gene (cox1) extracted from 2 cysticerci from the liver of *Ctenomys* sp. 1 (forward and reverse) of the liver from Terraplén Lagoon and, 2 polycephalic larvae, 1 from the liver and 1 from the pancreas (forward and reverse) of *Ctenomys* sp. 2 from Talagapa, each using the Wizard® Genomic DNA Purification Kit (A1120) following the manufacturer's instructions. The amplification and sequencing procedure follows Bagnato *et al*. (2022). Sequences were deposited in GenBank. Sequences from other *Versteria* species as well as other species of Taeniidae were taken from GenBank and compared using BLAST (https://blast.ncbi.nlm.nih.gov/Blast.cgi).

### Sequence alignment

Sequences obtained from cysticerci from *Ctenomys* sp. 1 and *Ctenomys* sp. 2 were aligned and compared with the cox1 sequences obtained from adults of *V. cuja* using Multalin software (available at http://www.sacs.ucsf.edu/cgi-bin/multalin.py).

### Histological study

A liver sample from *Ctenomys* sp. 1 and cysticerci were fixed in 4% formalin and preserved in 70% ethanol; the liver was separated for histology and cysticerci for definitive preparations. A lung sample from *Ctenomys* sp. 2 was separated for histology. Liver samples from *Ctenomys* sp. 1 and *C. haigi*, and lung samples from *Ctenomys* sp. 2 were dehydrated, embedded in paraffin and sectioned at 3–5 *μ*m following standard histological techniques. Sections were stained with haematoxylin–eosin (H&E), toluidine blue and Gomori's trichrome for morphological description. Other liver sections from *Ctenomys* sp. 1 were incubated separately with the primary antibodies pancytokeratin (Pk, dilution 1/250, Agilent, Z0622) to identify epithelial origin cells, and proliferating cell nuclear antigen (PCNA, 1/3000 dilution, Sigma-Aldrich, SAB2701819) to identify proliferating cells. All techniques used were based on standardized protocols of the *Laboratorio de Histología y Embriología Descriptiva, Experimental y Comparada, Facultad de Ciencias Veterinarias*, *Universidad Nacional de La Plata* (Acuña *et al*., 2021).

## Results

Cyclophyllidea van Beneden in Braun, 1900

Taeniidae Ludwig, 1886

***Versteria cuja* Bagnato, Gilardoni and Digiani, 2022**

### Description of metacestodes

**Fig. 1.**
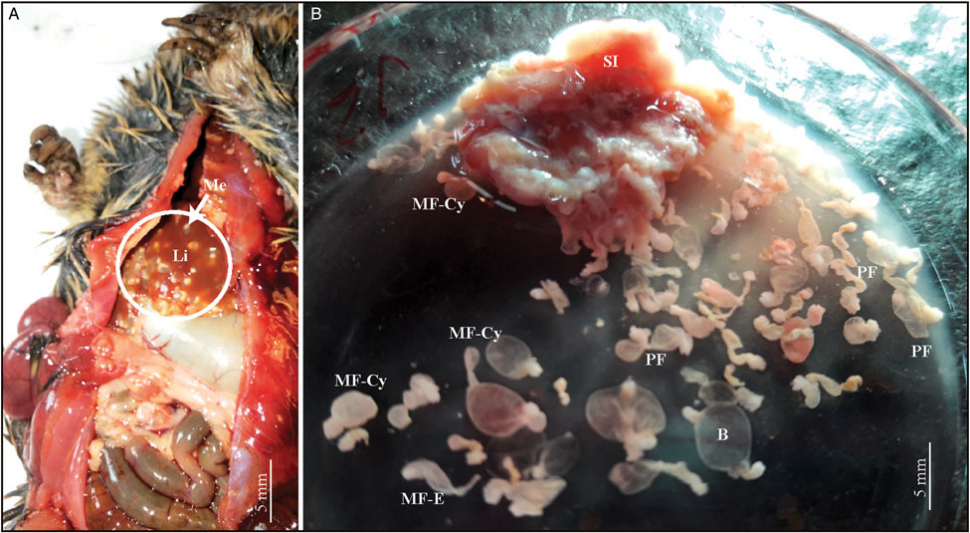
(A, B). Metacestodes of *Versteria cuja* Bagnato, Gilardoni and Digiani, 2022 (Cestoda: Taeniidae) invading organs in *Ctenomys* sp. 1 from Terraplén Lagoon, Chubut, Argentina. (A) Cysticercosis in liver, macroscopic view. (B) Mono- and polycephalic forms in small intestine, in fresh. B, bladder; Li, liver; Me, metacestodes; MF-Cy, monocephalic form-cysticercus; MF-E, monocephalic form-evaginated; PF, polycephalic forms; SI, small intestine.

**Fig. 2.**
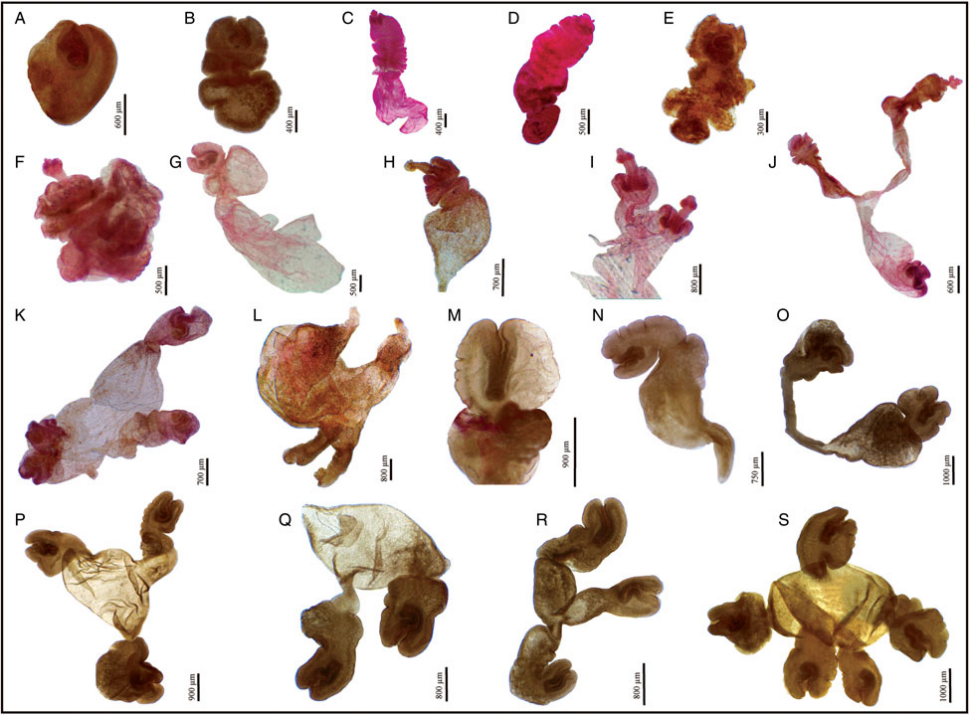
(A–J). Multiplicity of shapes and sizes of metacestodes of *Versteria cuja* Bagnato, Gilardoni and Digiani, 2022 (Cestoda: Taeniidae) found in *Ctenomys* sp. 1 from Terraplén Lagoon and *Ctenomys* sp. 2 from Talagapa, Chubut, Argentina. (A–H, M, N) Monocephalic forms. (I–L, O–S) Polycephalic forms. Note some forms with evaginated scoleces (C, D, H, I, J, L). (A–D, N, O–S) from the liver; (E, F) from the spleen; (G–L) from the small intestine; (M) from the pancreas. Stain: Langeron's carmine.

**Fig. 3.**
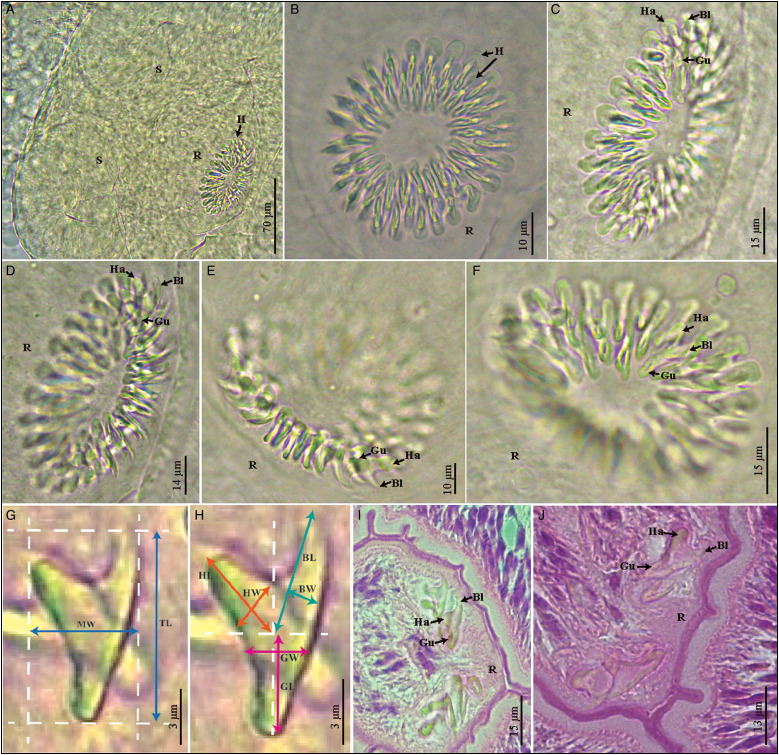
(A–J). Rostella and rostellar hooks from metacestodes of *Versteria cuja* Bagnato, Gilardoni and Digiani, 2022 (Cestoda: Taeniidae) found in *Ctenomys* sp. 1 and *Ctenomys* sp. 2 from Chubut, Argentina. (A–F) Rostella, showing the 2 crowns of alternate rostellar hooks of similar size, in different views, Hoyer's fluid. (G, H) Rostellar hooks, showing the 3 parts (handle, blade and guard) and measurements taken, lactoglycerol. (I, J) Rostellar hooks in histological sections, H&E. Bl, blade; BL, blade length; BW, blade width; Gu, guard; GL, guard length; GW, guard width; H, hooks, Ha, handle; HL, handle length; HW, handle width; MW, maximum width; R, rostellum; S, sucker; TL, total length.

Both mono- and polycephalic larvae were found in both intermediate hosts infecting several organs (Fig. 1). Both types of metacestodes exhibited a great variation in shapes (Fig. 2). The primary morphological characters allowing the association of these metacestodes to *V. cuja* were the rostellar hooks (number, size and shape); these are fully formed and represented by 3 typical parts: handle, blade and guard. The general measurements of 10 complete hooks of metacestodes from both intermediate hosts are: 13 (10–16) in total length, 8 (6–10) maximum width, handle 7 (5–10) in length by 4 (3–5) width, blade 6 (5–8) in length by 2 (2–3) width and guard 7 (5–8) in length by 3 (2–4) width (Fig. 3). Hooks are 40–48 in number, arranged in 2 alternating rows (Fig. 3).

*Monocephalic larvae* (Figs 1–3, Table 1, Supplementary Tables S1 and S2)

These forms corresponded to the cysticercus type, with one scolex per bladder. They were found mainly invading the liver, occupying the entire organ both superficially and deeply. Cysticerci also invaded the spleen, pancreas, small intestine and lungs, but in smaller numbers. Most larvae encountered had the typical cysticercus appearance with one scolex invaginated into the bladder. Some forms, however, showed evaginated scoleces. All scoleces observed exhibited 4 unarmed suckers and a rostellum bearing 2 rows of small hooks (Fig. 3).

Measurements provided are based on 66 metacestodes from different organs from both species of intermediate hosts (Supplementary Tables S1 and S2).

In *Ctenomys* sp. 1, cysticerci were found in the liver, spleen and small intestine and exhibited different shapes and sizes according to the organ infected. Those from the liver and spleen were smaller than those from the small intestine, which were the largest and had elongated bladders. The largest cysticercus was 8 mm in length by 2.75 mm wide (from the small intestine) and the smallest one (from the liver) measured 1.25 mm in length by 0.41 mm wide (Supplementary Table S1). Each cysticercus was characterized by a thin, transparent or whitish wall enclosing a fluid-filled bladder and a single small invaginated scolex. In mounted preparations, the bladders were collapsed.

In *Ctenomys* sp. 2, cysticerci were found in the liver, pancreas and lungs, enclosed in larger cysts containing 1–15 cysticerci. The cysticerci from the pancreas were smaller than those from the liver, which were the largest and had larger bladders. The largest cysticercus was 5.38 mm in length by 2.2 mm wide (from the liver) and the smallest one (from the pancreas) 2.38 in length by 0.88 mm wide (Supplementary Table S2). Each cysticercus had a thin, transparent or whitish wall enclosing a fluid-filled bladder and a single small invaginated scolex. The few larvae identified in the lungs were observed only in the histological sections.

*Polycephalic larvae* (Figs 1–3, Table 1, Supplementary Table S3)

Polycephalic larvae were found invading the first portion of the small intestine in *Ctenomys* sp. 1, and the liver and pancreas in *Ctenomys* sp. 2, the latter enclosed in cysts. Each cyst consisted of a capsule (probably originating from the connective tissue of the host) containing up to 15 larvae. The larvae from the small intestine were not observed inside a cyst; when the intestinal wall was opened, they all came out together in the Petri dish (see Fig. 1B). Polycephalic larvae presented a common bladder filled with fluid and containing a mean of 3 scoleces per bladder (Table 1). Different morphologies were observed: (1) dendritic or branched forms with a small to very small central bladder from which elongate stalks arose, each one bearing a scolex at the tip (mostly invaginated but sometimes evaginated) (Fig. 2I, J, O); (2) forms with a larger, common bladder, from which exogenous buds arose, each one bearing a scolex (Fig. 2K, L, P–S). In general, the mean length was higher in the metacestodes from *Ctenomys* sp. 1 (Table 1). The largest polycephalic metacestode from *Ctenomys* sp. 1 was 13.9 mm in length by 5.2 mm wide and the smallest one 3.0 mm in length by 0.9 mm wide. The largest form from *Ctenomys* sp. 2 was 9.23 mm in length by 6.75 mm wide (from the liver) and the smallest one (from the pancreas) 1.6 mm in length by 1.9 mm wide (Supplementary Table S3). The largest larva has a maximum of 5 scoleces arising from a central bladder.

Comparison of measurements between larvae from the 2 host species did not yield significant differences in diagnostic characters (scolex, rostellum, suckers); as there were few measurements of the different parts of the rostellar hooks, they could not be included in the ANOVA, but no differences were observed between both intermediate hosts. Only slight differences were found in the total larval length, length of the invaginated part (‘neck length’) and length of the exogenous buds in polycephalic larvae, differences which can probably be attributed to differences in the fixation method and to diverse degrees of regression of the central bladder, respectively.

### Taxonomic summary

Intermediate hosts: Tuco-tucos, *Ctenomys* sp. 1 and *Ctenomys* sp. 2 (Rodentia: Ctenomyidae).

Site of infection and abundance: In *Ctenomys* sp. 1: mainly in the liver (*n* = + 150), spleen (*n* = 8) and small intestine (*n* = 52). In *Ctenomys* sp. 2: liver (*n* = 15), pancreas (*n* = 30), lungs (*n* = 3, observed in histological sections).

Prevalence and intensity of infection: In *Ctenomys* sp. 1, *P* = 25% (1 out of 4 *Ctenomys* sp. 1 infected with +158 mono- and 52 polycephalic metacestodes). In *Ctenomys* sp. 2, one tuco-tuco with at least 48 mono- and polycephalic metacestodes.

Localities: Terraplén Lagoon (42.96°S, 71.49°W), near Los Alerces National Park, Chubut province, Argentina. Talagapa town (42.16°S, 68.17°W), Chubut province, Argentina.

Material deposited: From *Ctenomys* sp. 1: monocephalic and polycephalic forms, MLP-He 7988. From *Ctenomys* sp. 2: monocephalic and polycephalic forms, MLP-He 7989.

Host specimens deposited: LIEB-M-1727 (*Ctenomys* sp. 1, Terraplén Lagoon), LIEB-M-1763 (*Ctenomys* sp. 2, Talagapa).

### Molecular analysis

PCR amplification of cox1, mtDNA from cysticerci of *Versteria* from the liver of *Ctenomys* sp. 1 gave 2 products of 376 and 361 bp, respectively; and cysticerci of *Versteria* from the liver and pancreas of *Ctenomys* sp. 2 gave 2 products of 313 and 374 bp, respectively, of partial cox1 sequence. The cox1 sequences of cysticerci from *Ctenomy*s sp. 1, cysticerci from *Ctenomys* sp. 2 and the cox1 sequences of the adult worm from *G. cuja* (OL345569, OL345573 and OL345572) showed 100% similarity.

GenBank accession numbers: ON980784 and OP379709 (cox1, monocephalic forms from the liver of *Ctenomys* sp. 1); OP379295 (cox1, polycephalic form from the liver of *Ctenomys* sp. 2), OP379710 (cox1, polycephalic form from the pancreas of *Ctenomys* sp. 2).

**Fig. 4.**
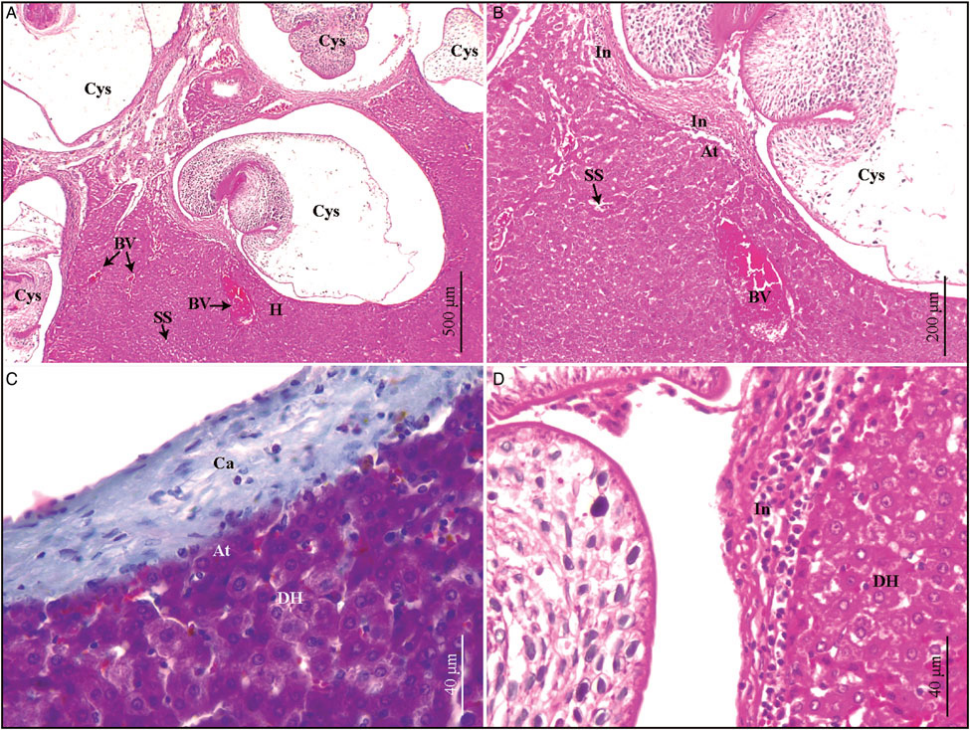
(A–D). Histological sections of *Ctenomys* sp.1 liver infected with *Versteria cuja* Bagnato, Gilardoni and Digiani, 2022. (A) Liver morphology altered by cysticerci. Congested blood vessels, hepatocytes lacking radial arrangement and dilated sinusoidal spaces are seen, haematoxylin–eosin (H&E). (B) Cysticercus surrounded by acidophilic capsule of connective tissue. Next to this area, infiltrate of immune cells: lymphocytes, macrophages and eosinophils. Hepatocytes adjacent to infiltrate and capsule are atrophied. Detail of the sinusoidal dilatation is also seen, H&E. (C) Detail of capsule circumscribing the cysticerci. Adjacent atrophied hepatocytes are observed. Other hepatocytes not atrophied although with degenerative changes (turbid tumefaction), Gomori's trichrome. (D) Degenerating hepatocytes with numerous cytoplasmic vacuoles and nuclear figures, H&E. At, atrophied hepatocytes; BV, blood vessels; Ca, capsule; Cys, cysticercus; DH, degenerating hepatocytes; H, hepatocytes; In, infiltrate; SS, sinusoidal spaces.

**Fig. 5.**
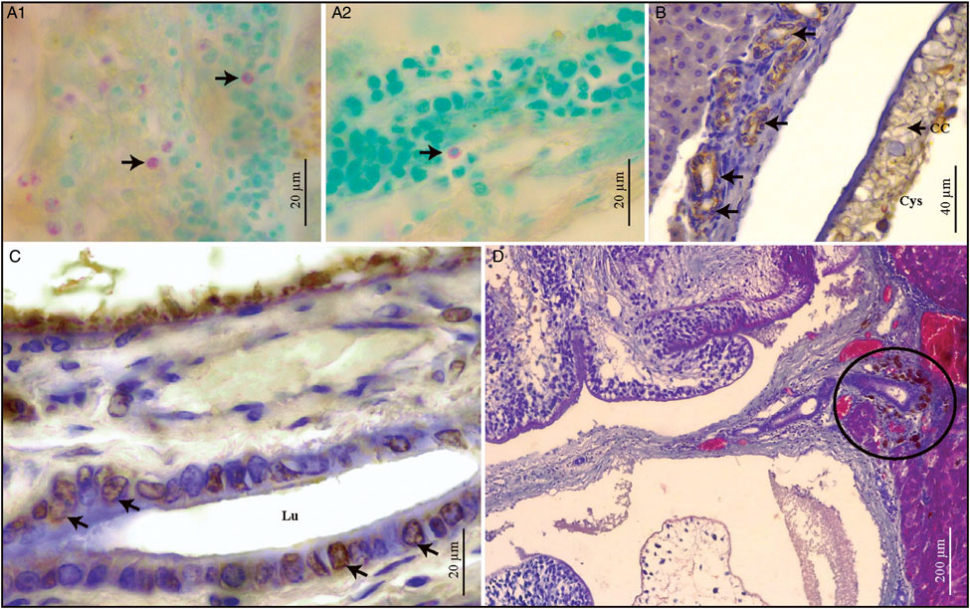
(A–D). Histological sections of *Ctenomys* sp.1 liver infected with *Versteria cuja* Bagnato, Gilardoni and Digiani, 2022. (A.1–A.2) Mast cells (arrows), cytoplasmatic and metachromatic granules are observed, Toluidine blue. (B) Multiple intact bile ducts are observed in the portal spaces, no alterations are observed in the lining epithelium (Pk+) (arrows), immunohistochemistry for pancytokeratin (Pk). (C) Proliferating epithelial cells (arrows) of the bile duct (PCNA+), immunohistochemistry for proliferating cell nuclear antigen (PCNA). (D) Macrophages with endocytosed haemosiderin (circle with black outline) adjacent to the bile ducts, Gomori's trichrome. CC, calcareous corpuscles; Cys, cysticercus; Lu, lumen.

### Histopathological description of Ctenomys sp. *1 liver* (Figs 4 and 5)

Cysticerci were observed in the hepatic parenchyma of *Ctenomys* sp. 1. The liver morphology was altered by the presence of cysticerci (Fig. 4A). Each cysticercus compressed the surrounding hepatocytes, altering their radial arrangement and causing atrophy. Congested blood vessels and dilated sinusoidal spaces were also observed (Fig. 4B). Degenerative changes (turbid tumefaction) were observed in the hepatocytes adjacent to the atrophied ones (Fig. 4C and D). Each cysticercus was surrounded by an acidophilic capsule of dense connective tissue, accompanied by an inflammatory infiltrate in which macrophages, lymphocytes and eosinophils predominated (Fig. 4D). In addition to lymphocytes and macrophages, mast cells with metachromatic granules were observed (Fig. 5A1–A2). Multiple bile ducts were observed intact, in the portal spaces, without morphological changes, but with proliferating cells (Pk and PCNA+) (Fig. 5B and C). Inflammatory cells including macrophages with endocytosed haemosiderin were observed adjacent to the bile ducts (Fig. 5D).

Comparing the parasitized liver of *Ctenomys* sp. 1 against a healthy liver (*C. haigi*), the latter showed hepatocytes polygonal shaped, acidophilic cytoplasm and 1–2 nuclei, arranged in trabeculae which radiated into the centrilobular vein. An additional difference was found in the number of bile ducts. Although in the healthy liver there was one bile duct per portal space, there was more than one bile duct in the parasitized liver (Fig. 5B).

### Histopathological description of *Ctenomys* sp. 2 lungs

**Fig. 6.**
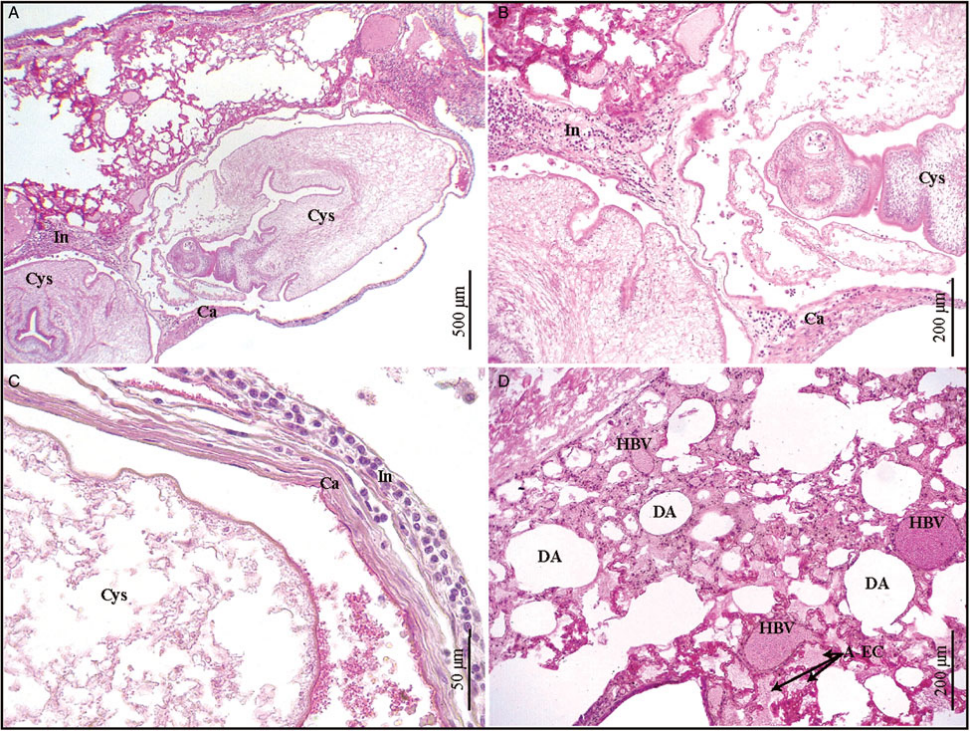
(A–D). Histological sections of *Ctenomys* sp. 2 lung infected with *Versteria cuja* Bagnato, Gilardoni and Digiani, 2022. (A) Two cysticerci surrounded by connective tissue capsule and inflammatory infiltrate in the lung parenchyma, haematoxylin–eosin (H&E). (B, C) Magnifications of (A), H&E. (B) Scolex. (C) Posterior end of the cysticercus. (D) Hyperaemic blood vessels, dilated alveoli and alveoli with eosinophilic content, H&E. A-EC, alveoli with eosinophilic content; Ca, capsule; Cys, cysticercus; DA, dilated alveoli; HBV, hyperaemic blood vessels; In, infiltrate.

Cysticerci were observed in the lung parenchyma of *Ctenomys* sp. 2. The lung morphology was altered by the presence of cysticerci (Fig. 6). Each cysticercus was associated with an inflammatory infiltrate and surrounded by a connective tissue capsule (Fig. 6A–C). Several pulmonary alveoli were observed to be dilated. Other pulmonary alveoli contained eosinophilic material, evidence of oedema, in the lumen. Some blood vessels were observed to be hyperaemic (Fig. 6D).

## Discussion

The similarity (in number, size and shape) of the rostellar hooks of the metacestodes found in *Ctenomys* sp. 1 and *Ctenomys* sp. 2 to those observed in the adult cestodes from the lesser grison in the same area (Bagnato *et al*., 2022) lead us to suspect that they could be the metacestodes of *V. cuja*. This similarity could be observed in definitive and transitory preparations as well as in histological sections. Hooks' dimensions of metacestodes were 13 (10–16) in length by 8 (6–10) wide, whereas in the adult tapeworm these were (12–17) in length by (6–10) wide. The total number of hooks was 40–48 in the metacestodes and 48 in the adults (Bagnato *et al*., 2022). Based on this first evidence, we performed the molecular analysis, which finally confirmed the identity of *V. cuja* in the 2 intermediate host species. The 100% similarity between the cox1 sequences of cysticerci from *Ctenomys* sp. 1, polycephalic larvae from *Ctenomys* sp. 2 and the adult worm from the lesser grison allowed confirmation, for *V. cuja*, of a 2-host life cycle with the subterranean rodents (*Ctenomys* sp. 1 and *Ctenomys* sp. 2) as intermediate hosts and the lesser grison *G. cuja* as definitive host in Chubut province, Argentina. The metacestodes were found invading different organs and showed a multiplicity of shapes and sizes which can be summarized primarily into 2 types: monocephalic forms (the typical cysticercus) with one scolex per bladder, and polycephalic forms in which 2 or more scoleces arose from a common bladder. In addition, within both types most larvae showed typically invaginated scoleces; however, a small proportion of larvae showed evaginated scoleces.

‘Polycephalic’ larvae were defined by Hoberg *et al*. (2000) as larvae in which 2 or more scoleces are borne on elongate stalks that arise by exogenous budding from a central bladder that later regresses (Hoberg *et al*., 2000; Chervy, 2002). These larvae were considered to be derived forms with respect to the cysticercus and characteristic of some taeniid species such as *Taenia selousi* Mettrick, 1962, *Taenia endothoracicus* (Kirschenblatt, 1948) and *Taenia twitchelli* Schwartz, 1927 but also of *T. mustelae sensu* Freeman (1956) (Hoberg *et al*., 2000). Rossin *et al*. (2010) found both mono- and polycephalic larvae that were assigned to *Taenia talicei* (Dollfus,1960) in the abdominal cavity of 2 *Ctenomys* species (*C. talarum* Thomas and *C. australis* Rusconi) from Buenos Aires province, Argentina (Rossin *et al*., 2010).

The multiplicity of forms encountered among the polycephalic larvae of *V. cuja* could be explained by the different degrees of regression of the central bladder. As mentioned above, most larvae showed typically invaginated scoleces; however, a small proportion showed evaginated scoleces. Moreover, some polycephalic larvae exhibited the 2 conditions simultaneously (see Fig. 2J and L).

The significance of the evagination and invagination of the scolex, at least within the Taeniidae, remains unclear (see Świderski *et al*., 2007). Even when taeniids are characterized by scolex morphogenesis in an invaginated condition, exceptions may appear. As summarized by Loos-Frank (2000) and Świderski *et al*. (2007) among taeniid species with polycephalic larvae, *Taenia crassiceps* (Zeder, 1800), *T. endothoracicus*, *T. mustelae*, *Taenia parva* Baer, 1926 and *T. twitchelli* have post-embryonic larvae with scoleces invaginated. In contrast, evaginated scoleces have been reported in *Taenia krepkogorski* (Schulz and Landa, 1934) and *T. selousi*.

On the other hand, the evagination of the scoleces of other taeniid species and genera may be induced in *in vitro* conditions. For example, Maimaitizunong *et al*. (2022) demonstrated that a brief stimulation with water (2 times of 20s) prior to *in vitro* cultivation of protoscoleces of *E. granulosus* and *E. multilocularis* increased the evagination process, resulting in 85–90% of evaginated protoscoleces.

Thus, our observations of a few evaginated scoleces among the isolated larvae could be related to the medium in which cysticerci weremanipulated. Cysticerci from *Ctenomys* sp. 1, which exhibited both invaginated and evaginated scoleces, were washed in physiological solution prior to their fixation in 4% formalin, while cysticerci from *Ctenomys* sp. 2 (all invaginated forms) were placed directly in 96% ethanol. The presence of one larva with an evaginated scolex surrounded by the host capsule within the lung (Fig. 6) is, on the contrary, more difficult to explain.

The larval stages of *V. cuja* are largely similar to those described by Freeman (1956) for ‘*Taenia mustelae*’ from the USA in naturally and experimentally infected intermediate hosts. Freeman (1956) also found infection in various organs, and monocephalic and polycephalic forms coexisting in a same individual host. The rostellar hooks of the metacestodes of *V. cuja* had similar shape and size than those of the North American species, having a short and sharply curved blade; short, straight to sinuous handle and long stout guard. The handle ended in a stout bulb and the posterior edge was frequently folded, in a similar way to that described by Freeman (1956). The hooks of metacestodes of *V. cuja* ranged in total length from 10 to 16 *vs* 14 to 20 in those studied by Freeman (1956).

It is worth noting that studies on the life cycle of *V. mustelae* (=*T. mustelae*) in the Palaearctic region (Murai, 1981; Iwaki *et al*., 1996) reported only monocephalic cysticerci, whereas in the Nearctic ‘*T. mustelae*’ (as it appears in older reports) mono- and polycephalic metacestodes simultaneously occur (Loos-Frank, 2000). It seems then that a life cycle with mono- and polycephalic larvae is another feature characterizing a distinct Nearctic lineage of *Versteria* (different from *V. mustelae*), besides the molecular differences and the zoonotic potential (Skinker, 1935; Locker, 1955; Freeman, 1956; McKeever and Henry, 1971; Niedringhaus *et al*., 2021).

Indeed, Niedringhaus *et al*. (2021) described a case of fatal infection by *Versteria* in a muskrat *Ondatra zibethicus* from Pennsylvania, USA. Multiple organs of the muskrat were infected, harbouring dozens of cysts lined by a thin, fibrous capsule and containing solid-bodied larvae with up to 4 invaginated, small scoleces bearing short rostellar hooks (up to 11 in length), arranged in 2 rows. These larvae were named by Niedringhaus *et al*. (2021) as ‘polycephalic cysticerci’. Its genetic characterization confirmed the cestode as belonging to the lineage known as ‘zoonotic’ *Versteria* sp., known from several cases of metacestodiasis in humans and other primates, probably caused by the accidental ingestion of the eggs (see Deplazes *et al*., 2019).

The similarities (in morphological characteristics and type of infection) between the metacestodes of *V. cuja* in ctenomyids from Argentina and those reported by Freeman (1956) and Niedringhaus *et al*. (2021) agree with the idea that *V. cuja* and the North American species (*T. mustelae sensu* Freeman (1956)/*Versteria* sp. *sensu* modern authors) are closely related (Bagnato *et al*., 2022). This means that *V. cuja* could be a new, potentially zoonotic agent in this region of the continent. Indeed, versteriosis due to accidental ingestion by humans of eggs of this species remains hypothetical but should not be disregarded.

Niedringhaus *et al*. (2021) also provided a histopathological description of the lesions caused by the metacestodes of *Versteria* sp. in the liver of *O. zibethicus*. In their study, post-mortem examination revealed widespread tissue loss and replacement by solid-bodied cestode larvae with minimal adjacent inflammation in many visceral organs.

Similarly to the histological findings reported by Niedringhaus *et al*. (2021) for *O. zibethicus*, the parasitized liver of *Ctenomys* sp. 1 showed compression of hepatocytes and changes in their arrangement, congested blood vessels and inflammatory infiltrate with predominance of mononuclear lymphocytes. Regarding the histopathological aspect, it is assumed that the presence of the cysticerci not only altered the arrangement and shape of the hepatocytes, but could also have caused a reduction in blood flow and thus, the supply of nutrients, including oxygen, which would have produced degeneration in the hepatocytes adjacent to the atrophied ones. The presence of the capsule as well as the immune cells of the inflammatory infiltrate, corresponding to a parasitic-type infection, would be due to the response of the host's immune system to eliminate the agent. An additional difference with the non-parasitized liver was found in the number of bile ducts. Although in the healthy liver there was one bile duct per portal space, there were approximately 7 bile ducts in the parasitized liver. It is hypothesized that this may be due to the reactivity of the organ by chronicity of the infection (Weiss *et al*., 1997). Hepatitis in the portal space has been associated with biliary hyperplasia in other species (Weiss *et al*., 1997; Lefkowitch, 2005; Fox *et al*., 2009; Portas and Taylor, 2015).

Niedringhaus *et al*. (2021) found heavily parasitized lungs with cysticerci cysts, while the lungs of *Ctenomys* sp. 2 were little parasitized; only 3 cysticerci were observed in the lung parenchyma. The 3 cysts found were observed in the histological sections. Superficially, cysts were seen in the lung as white tumours, and because of this the lung was fixed for histology, but the parasite was not suspected to be the aetiological agent. Despite this variation, there were similar morphological changes such as connective tissue encapsulation and inflammatory response. Other morphological changes such as dilated alveoli and alveoli with eosinophilic content (oedema) were also observed. These 2 histopathological characteristics are probably related to the reduced expansion capacity of the lungs due to the presence of parasitic cysts. In addition to liver, gastrointestinal tract and lungs, Niedringhaus *et al*. (2021) found other organs infected such as kidneys, ovaries and brain, which was not the case in *Ctenomys* spp.

This study is the first report of a natural life cycle of a species of *Versteria* in South America. It integrates taxonomic, morphological, ecological and histopathological aspects of the interaction between a mustelid species, a subterranean rodent and a parasitic tapeworm, with potential transmission to humans.

## Data Availability

Four of 8 sequences used in the analyses performed here were submitted and are available at GenBank under accession numbers ON980784, OP379709, OP379710 and OP379295.

## Supplementary material

For supplementary material accompanying this paper visit https://doi.org/10.1017/S0031182023000215. click here to view supplementary material
