# Supplementary material for: Natural life cycle of Versteria cuja (Taeniidae) in Argentina and histopathology of metacestodiasis in intermediate hosts
Source: Parasitology. 2023 Mar 8;150(6):488–97. doi: 10.1017/S0031182023000215 (PMC10260299; doi:10.1017/S0031182023000215)
Supplement: Supplementary file 1 [file S0031182023000215sup.zip › S0031182023000215sup001.docx]

**Supplementary material Table S1**. Measurements (in micrometers) of monocephalic larvae of *Versteria cuja* from *Ctenomys* sp. 1, Chubut province, Argentina.

| Monocephalic larvae | *Ctenomys* sp. 1- Terrapén Lagoon | | | | | | | | | | | | | | | | | | | |
| --- | --- | --- | --- | --- | --- | --- | --- | --- | --- | --- | --- | --- | --- | --- | --- | --- | --- | --- | --- | --- |
| Metacestode type | Cysticercus | | | | Cysticercus | | | | Cysticercus | | | | “Evaginated forms” | | | | “Evaginated forms” | | | |
| Site of infection | Liver | | | | Spleen | | | | Small intestine | | | | Liver | | | | Small intestine | | | |
|  | Mean | Min | Max | n | Mean | Min | Max | n | Mean | Min | Max | n | Mean | Min | Max | n | Mean | Min | Max | n |
| Total length | 2,354 | 1,250 | 4,300 | 21 | 1,557 | 1,250 | 1,880 | 3 | 4,476 | 1,880 | 8,000 | 22 | 3,100 | 2,100 | 3,900 | 4 | 3,777 | 2,250 | 5,380 | 3 |
| Maximum width | 1,093 | 410 | 1,630 | 21 | 973 | 700 | 1,320 | 3 | 1,533 | 800 | 2,750 | 22 | 1,138 | 900 | 1,350 | 4 | 1,103 | 630 | 1,380 | 3 |
| Bladder length | - | - | - | - | - | - | - | - | - | - | - | - | 1,531 | 875 | 2,250 | 4 | 2,547 | 1,380 | 4,130 | 3 |
| Bladder width | - | - | - | - | - | - | - | - | - | - | - | - | 1,131 | 875 | 1,350 | 4 | 1,103 | 630 | 1,380 | 3 |
| Scolex length | 337 | 143 | 730 | 14 | 237 | 210 | 250 | 3 | 265 | 140 | 400 | 13 | 179 | 125 | 220 | 4 | 260 | 200 | 350 | 3 |
| Scolex width | 305 | 108 | 500 | 14 | 257 | 220 | 300 | 3 | 302 | 200 | 450 | 13 | 183 | 90 | 240 | 4 | 257 | 220 | 300 | 3 |
| Rostellum diameter | 51 | 30 | 80 | 3 | - | 20 | 40 | 2 | 52 | 20 | 90 | 8 | 119 | 37 | 250 | 3 | 40 | 30 | 50 | 3 |
| Suckers diameter | 89 | 70 | 110 | 7 | - | 80 | 80 | 1 | 118 | 60 | 200 | 10 | 93 | 70 | 110 | 3 | 117 | 90 | 150 | 3 |
| Neck length | - | - | - | - | - | - | - | - | - | - | - | - | - | 200 | 200 | 1 | 323 | 150 | 520 | 3 |
| Neck width | - | - | - | - | - | - | - | - | - | - | - | - | - | 90 | 90 | 1 | 233 | 130 | 330 | 3 |
| Nº hooks | - | 40 | 40 | 1 | - | - | - | - | - | 44 | 44 | 1 | - | 48 | 48 | 1 | - | - | - | - |
| Hook length | 10 | 10 | 10 | 7 | - | - | - | - | - | - | - | - | 10 | 8 | 11 | 6 | - | - | - | - |
| Hook width | 4 | 3 | 4 | 5 | - | - | - | - | **-** | - | - | - | - | 5 | 5 | 1 | - | - | - | - |

Abbreviations: Max, maximum; Min, minimum; n, number of measurements.
