# Supplementary material for: Natural life cycle of Versteria cuja (Taeniidae) in Argentina and histopathology of metacestodiasis in intermediate hosts
Source: Parasitology. 2023 Mar 8;150(6):488–97. doi: 10.1017/S0031182023000215 (PMC10260299; doi:10.1017/S0031182023000215)
Supplement: Supplementary file 1 [file S0031182023000215sup.zip › S0031182023000215sup002.docx]

**Supplementary material Table S2.** Measurements (in micrometers) of monocephalic larvae of *Versteria cuja* from *Ctenomys* sp. 2, Chubut province, Argentina.

| Monocephalic larvae | *Ctenomys* sp. 2- Talagapa | | | | | | | | |
| --- | --- | --- | --- | --- | --- | --- | --- | --- | --- |
|  |  |  |  |  |  |  |  |  |  |
| Metacestode type | Cysticercus | | | | Cysticercus | | | | “Evaginated forms” |
| Site of infection | Liver | | | | Pancreas | | | | Pancreas |
|  | Mean | Min | Max | n | Mean | Min | Max | n | n=1 |
| Total length | 3,710 | 2,750 | 5,380 | 3 | 3,472 | 2,380 | 4,500 | 6 | 1,650 |
| Maximum width | 1,593 | 1,200 | 2,200 | 3 | 1,417 | 880 | 1,750 | 6 | 960 |
| Bladder length | - | - | - | - | - | - | - | - | 370 |
| Bladder width | - | - | - | - | - | - | - | - | 1,020 |
| Scolex length | 177 | 130 | 200 | 3 | 175 | 130 | 210 | 6 | 150 |
| Scolex width | 280 | 240 | 300 | 3 | 258 | 240 | 300 | 6 | 250 |
| Rostellum diameter | - | 40 | 60 | 2 | 60 | 60 | 60 | 3 | 50 |
| Suckers diameter | 117 | 110 | 120 | 3 | 125 | 70 | 200 | 6 | 100 |
| Neck length | 583 | 500 | 700 | 3 | 602 | 500 | 630 | 5 | 600 |
| Neck width | 293 | 240 | 390 | 3 | 354 | 250 | 500 | 5 | 500 |
| Nº hooks | - | - | - | - | - | - | - | - | - |
| Hook length | - | - | - | - | - | - | - | - | 11 (n=8) |
| Hook width | - | - | - | - | - | - | - | - | - |

Abbreviations: Max, maximum; Min, minimum; n, number of measurements.
