# Supplementary material for: Natural life cycle of Versteria cuja (Taeniidae) in Argentina and histopathology of metacestodiasis in intermediate hosts
Source: Parasitology. 2023 Mar 8;150(6):488–97. doi: 10.1017/S0031182023000215 (PMC10260299; doi:10.1017/S0031182023000215)
Supplement: Supplementary file 1 [file S0031182023000215sup.zip › S0031182023000215sup003.docx]

**Supplementary material Table S3.** Measurements (in micrometers) of polycephalic larvae of *Versteria cuja* from *Ctenomys* sp. 1 and *Ctenomys* sp. 2, Chubut province, Argentina.

| Polycephalic larvae | *Ctenomys* sp. 1 | | | | *Ctenomys* sp. 2 | | | | | | | |
| --- | --- | --- | --- | --- | --- | --- | --- | --- | --- | --- | --- | --- |
| Site of infection | Small intestine | | | | Liver | | | | Pancreas | | | |
|  | Mean | Min | Max | n | Mean | Min | Max | n | Mean | Min | Max | n |
| Number scolices per bladder | 3 | 2 | 4 | 11 | 3 | 1 | 5 | 11 | 2 | 1 | 3 | 8 |
| Total length | 8,322 | 3,000 | 13,900 | 11 | 5,510 | 3,130 | 9,230 | 11 | 3,484 | 1,600 | 6,750 | 10 |
| Maximum total width | 2,518 | 900 | 5,200 | 11 | 3,357 | 1,750 | 6,750 | 11 | 2,777 | 1,900 | 4,630 | 10 |
| Exogenous buds length | 4,258 | 1,000 | 9,750 | 34 | 1,755 | 1,000 | 3,113 | 23 | 2,639 | 1,380 | 5,130 | 31 |
| Exogenous buds width | 1,039 | 500 | 1,880 | 34 | 1,121 | 700 | 2,130 | 23 | 1,441 | 877 | 2,250 | 31 |
| Central bladder length | 1,553 | 380 | 4,000 | 4 | 1,732 | 880 | 2,250 | 5 | 2,157 | 1,380 | 3,050 | 6 |
| Central bladder width | 783 | 600 | 1,250 | 4 | 1,754 | 380 | 2,630 | 5 | 2,485 | 880 | 3,630 | 6 |
| Scolex length | 264 | 203 | 330 | 23 | 168 | 118 | 246 | 29 | 170 | 130 | 235 | 19 |
| Scolex width | 288 | 200 | 385 | 23 | 288 | 201 | 406 | 29 | 252 | 205 | 290 | 19 |
| Rostellum diameter | 58 | 30 | 80 | 19 | 50 | 47 | 53 | 4 | 42 | 25 | 60 | 5 |
| Sucker diameter | 113 | 90 | 130 | 17 | 135 | 104 | 188 | 29 | 120 | 95 | 160 | 19 |
| Neck length | 555 | 358 | 900 | 23 | 770 | 620 | 962 | 29 | 683 | 440 | 940 | 17 |
| Neck width | 296 | 190 | 440 | 23 | 394 | 276 | 570 | 29 | 352 | 200 | 635 | 17 |
| Nº hooks | 44 | 44 | 44 | 3 | - | - | - | - | - | - | - | - |
| Hook L | 14 | 11 | 18 | 16 | 18 | 15 | 21 | 5 | - | - | - | - |
| Hook W | 10 | 9 | 10 | 14 | 13 | 12 | 14 | 4 | - | - | - | - |

Abbreviations: Max, maximum; Min, minimum; n, number of measurements.
